# Supplementary material for: Boreal marine fauna from the Barents Sea disperse to Arctic Northeast Greenland
Source: Sci Rep. 2019 Apr 9;9:5799. doi: 10.1038/s41598-019-42097-x (PMC6456728; doi:10.1038/s41598-019-42097-x)
Supplement: Supplementary file 1 — Supplementary information [file 41598_2019_42097_MOESM1_ESM.pdf]

SUPPLEMENTARY INFORMATION

Boreal marine fauna from the Barents Sea disperse to Arctic Northeast Greenland

Adam J. Andrews, Jørgen S. Christiansen, Shripathi Bhat, Arve Lynghammar, Jon-Ivar Westgaard, Christophe Pampoulie, Kim Præbel

Supplementary Text S1: Assignment membership probabilities

STRUCTURE and *snapclust* membership probabilities (q) for cod (Table S1.1), redfish (Table S1.2) and shrimp (Table S1.3) specimens caught off Northeast Greenland are shown, and summarised with the most probable assignment origin indicated for both methods.

Table S1.1. STRUCTURE and *snapclust* identified cod population of origin and membership probabilities (q) for Northeast Greenland (NEG) cod individuals using all loci. Bold typeface indicates the q values supporting the most probable origin of each individual. For abbreviations refer to Table S4.

Sample	STRUCTURE origin	*snapclust* origin	STRUCTURE q values			*snapclust* q values		
			ICE	NEAC	NCC	ICE	NEAC	NCC
NEG Cod 1	NEAC	NEAC	0.133	**0.852**	0.015	0.230	**0.630**	0.140
NEG Cod 2	NEAC	NEAC	0.074	**0.925**	0.001	0.056	**0.943**	0.001
NEG Cod 3	NEAC	NEAC	0.158	**0.839**	0.003	0.388	**0.601**	0.011
NEG Cod 4	NEAC	NEAC	0.148	**0.848**	0.003	0.380	**0.605**	0.015
NEG Cod 5	NEAC	NEAC	0.063	**0.935**	0.002	0.092	**0.887**	0.021
NEG Cod 6	NEAC	NEAC	0.160	**0.835**	0.005	0.373	**0.620**	0.007
NEG Cod 7	NEAC	NEAC	0.144	**0.840**	0.016	0.284	**0.631**	0.085
NEG Cod 8	NEAC	NEAC	0.171	**0.825**	0.004	0.466	**0.520**	0.014
NEG Cod 9	NEAC	ICE	0.188	**0.802**	0.010	**0.557**	0.326	0.117
NEG Cod 10	NEAC	NEAC	0.038	**0.962**	0.000	0.036	**0.962**	0.002

Table S1.2. STRUCTURE and *snapclust* identified redfish population of origin and membership probabilities (*q*) for Northeast Greenland (NEG) redfish individuals using all loci. Bold typeface indicates the *q* values supporting the most probable origin of each individual. For abbreviations refer to Table S4.

Sample	STRUCTURE origin	*snapclust* origin	STRUCTURE *q* values			*snapclust q* values		
			EGS	IDP	NSH	EGS	IDP	NSH
NEG Redfish 1	NSH	NSH	0.044	0.000	**0.956**	0.217	0.001	**0.782**
NEG Redfish 2	NSH	NSH	0.000	0.000	**1.000**	0.001	0.016	**0.983**
NEG Redfish 3	NSH	NSH	0.003	0.002	**0.995**	0.036	0.056	**0.908**
NEG Redfish 4	NSH	NSH	0.001	0.000	**0.999**	0.004	0.001	**0.995**
NEG Redfish 5	EGS	EGS	**0.870**	0.052	0.079	**0.553**	0.226	0.221
NEG Redfish 6	NSH	NSH	0.004	0.000	**0.996**	0.044	0.000	**0.956**
NEG Redfish 7	NSH	NSH	0.000	0.000	**1.000**	0.001	0.000	**0.999**
NEG Redfish 8	NSH	NSH	0.000	0.000	**1.000**	0.001	0.000	**0.999**
NEG Redfish 9	NSH	NSH	0.000	0.000	**1.000**	0.001	0.000	**0.999**
NEG Redfish 10	NSH	NSH	0.000	0.000	**1.000**	0.001	0.000	**0.999**
NEG Redfish 11	NSH	NSH	0.000	0.000	**1.000**	0.000	0.000	**1.000**
NEG Redfish 12	NSH	NSH	0.000	0.000	**1.000**	0.012	0.001	**0.987**
NEG Redfish 13	EGS	EGS	**0.919**	0.000	0.081	**0.990**	0.000	0.010
NEG Redfish 14	NSH	NSH	0.027	0.002	**0.971**	0.017	0.032	**0.801**
NEG Redfish 15	NSH	NSH	0.003	0.000	**0.997**	0.023	0.000	**0.977**
NEG Redfish 16	NSH	NSH	0.003	0.001	**0.996**	0.031	0.061	**0.908**
NEG Redfish 17	NSH	NSH	0.000	0.000	**1.000**	0.001	0.000	**0.999**
NEG Redfish 18	NSH	NSH	0.000	0.000	**1.000**	0.001	0.001	**0.998**
NEG Redfish 19	NSH	NSH	0.001	0.000	**0.999**	0.002	0.000	**0.998**
NEG Redfish 20	NSH	NSH	0.005	0.000	**0.995**	0.063	0.000	**0.937**
NEG Redfish 21	NSH	NSH	0.000	0.000	**1.000**	0.009	0.000	**0.991**
NEG Redfish 22	EGS	EGS	**0.746**	0.001	0.253	**0.953**	0.002	0.045
NEG Redfish 23	NSH	NSH	0.001	0.000	**0.999**	0.023	0.003	**0.974**
NEG Redfish 24	NSH	NSH	0.001	0.000	**0.999**	0.033	0.003	**0.964**
NEG Redfish 25	NSH	NSH	0.000	0.000	**1.000**	0.001	0.000	**0.999**
NEG Redfish 26	NSH	NSH	0.000	0.000	**1.000**	0.009	0.000	**0.991**
NEG Redfish 27	NSH	NSH	0.000	0.000	**1.000**	0.001	0.000	**0.999**
NEG Redfish 28	NSH	NSH	0.006	0.000	**0.994**	0.048	0.000	**0.952**
NEG Redfish 29	NSH	NSH	0.000	0.000	**1.000**	0.005	0.000	**0.995**
NEG Redfish 30	NSH	NSH	0.000	0.000	**1.000**	0.001	0.001	**0.998**
NEG Redfish 31	NSH	NSH	0.000	0.000	**1.000**	0.007	0.000	**0.993**

NEG Redfish 32	NSH	NSH	0.000	0.000	**1.000**	0.002	0.000	**0.998**
NEG Redfish 33	NSH	NSH	0.000	0.000	**1.000**	0.000	0.000	**0.999**
NEG Redfish 34	NSH	NSH	0.008	0.000	**0.992**	0.076	0.000	**0.924**
NEG Redfish 35	NSH	NSH	0.009	0.001	**0.990**	0.028	0.024	**0.948**
NEG Redfish 36	NSH	NSH	0.000	0.000	**1.000**	0.004	0.000	**0.996**
NEG Redfish 37	NSH	NSH	0.000	0.000	**1.000**	0.026	0.003	**0.971**
NEG Redfish 38	NSH	NSH	0.000	0.000	**1.000**	0.002	0.000	**0.998**
NEG Redfish 39	NSH	NSH	0.000	0.000	**1.000**	0.001	0.000	**0.999**
NEG Redfish 40	NSH	EGS	0.120	0.000	**0.880**	**0.693**	0.000	0.307
NEG Redfish 41	NSH	NSH	0.022	0.000	**0.978**	0.023	0.000	**0.977**
NEG Redfish 42	NSH	NSH	0.004	0.000	**0.996**	0.176	0.005	**0.819**
NEG Redfish 43	NSH	NSH	0.000	0.000	**1.000**	0.000	0.000	**1.000**
NEG Redfish 44	NSH	NSH	0.000	0.000	**1.000**	0.000	0.001	**0.999**
NEG Redfish 45	NSH	NSH	0.000	0.000	**1.000**	0.001	0.000	**0.999**
NEG Redfish 46	NSH	NSH	0.028	0.000	**0.971**	0.127	0.002	**0.871**
NEG Redfish 47	NSH	NSH	0.000	0.000	**1.000**	0.012	0.000	**0.988**
NEG Redfish 48	NSH	NSH	0.001	0.002	**0.997**	0.005	0.045	**0.950**
NEG Redfish 49	NSH	NSH	0.001	0.000	**0.999**	0.007	0.000	**0.993**
NEG Redfish 50	NSH	NSH	0.000	0.000	**1.000**	0.000	0.001	**0.999**
NEG Redfish 51	NSH	NSH	0.000	0.000	**1.000**	0.000	0.001	**0.999**
NEG Redfish 52	NSH	NSH	0.000	0.000	**1.000**	0.001	0.000	**0.999**
NEG Redfish 53	NSH	NSH	0.004	0.000	**0.996**	0.175	0.000	**0.825**
NEG Redfish 54	NSH	NSH	0.000	0.000	**1.000**	0.000	0.000	**1.000**
NEG Redfish 55	NSH	NSH	0.000	0.000	**1.000**	0.000	0.000	**1.000**
NEG Redfish 56	NSH	NSH	0.000	0.000	**1.000**	0.285	0.000	**0.715**
NEG Redfish 57	NSH	NSH	0.000	0.000	**1.000**	0.000	0.000	**1.000**
NEG Redfish 58	NSH	NSH	0.002	0.000	**0.998**	0.107	0.001	**0.892**
NEG Redfish 59	NSH	NSH	0.000	0.000	**1.000**	0.000	0.001	**0.999**
NEG Redfish 60	NSH	NSH	0.001	0.000	**0.999**	0.025	0.000	**0.975**
NEG Redfish 61	NSH	NSH	0.000	0.000	**1.000**	0.000	0.004	**0.996**
NEG Redfish 62	NSH	NSH	0.000	0.000	**1.000**	0.016	0.010	**0.974**
NEG Redfish 63	NSH	NSH	0.000	0.000	**1.000**	0.007	0.000	**0.993**
NEG Redfish 64	NSH	NSH	0.000	0.000	**1.000**	0.000	0.000	**1.000**

Table S1.3. STRUCTURE and *snapclust* identified shrimp population of origin and membership probabilities (*q*) for Northeast Greenland (NEG) shrimp individuals using all loci. Bold typeface indicates the *q* values supporting the most probable origin of each individual. For abbreviations refer to Table S4.

Sample	STRUCTURE origin	*snapclust* origin	STRUCTURE *q* values				*snapclust q* values			
			ICE	JMA	SPW	NOR	ICE	JMA	SPW	NOR
NEG Shrimp 1	SPW	ICE	0.027	0.027	**0.938**	0.008	**0.451**	0.439	0.096	0.014
NEG Shrimp 2	SPW	SPW	0.008	0.000	**0.976**	0.016	0.063	0.012	**0.751**	0.175
NEG Shrimp 3	SPW	SPW	0.008	0.015	**0.977**	0.000	0.301	0.253	**0.445**	0.002
NEG Shrimp 4	SPW	NOR	0.003	0.02	**0.914**	0.063	0.010	0.168	0.207	**0.615**
NEG Shrimp 5	SPW	SPW	0.015	0.001	**0.982**	0.002	0.197	0.022	**0.772**	0.009
NEG Shrimp 6	SPW	SPW	0.007	0.008	**0.962**	0.023	0.135	0.132	**0.534**	0.200
NEG Shrimp 7	SPW	NOR	0.006	0.000	**0.979**	0.015	0.159	0.009	0.257	**0.575**
NEG Shrimp 8	SPW	SPW	0.011	0.001	**0.983**	0.005	0.169	0.018	**0.715**	0.098
NEG Shrimp 9	SPW	SPW	0.004	0.000	**0.996**	0.000	0.028	0.012	**0.958**	0.001
NEG Shrimp 10	SPW	SPW	0.007	0.000	**0.987**	0.006	0.137	0.001	**0.805**	0.057
NEG Shrimp 11	SPW	SPW	0.002	0.013	**0.967**	0.018	0.008	0.349	**0.584**	0.058
NEG Shrimp 12	SPW	SPW	0.000	0.000	**0.998**	0.002	0.001	0.003	**0.967**	0.028
NEG Shrimp 13	SPW	SPW	0.000	0.001	**0.999**	0.000	0.000	0.098	**0.900**	0.001
NEG Shrimp 14	SPW	SPW	0.001	0.000	**0.998**	0.001	0.002	0.002	**0.991**	0.005
NEG Shrimp 15	SPW	SPW	0.005	0.001	**0.990**	0.004	0.023	0.026	**0.832**	0.119
NEG Shrimp 16	SPW	ICE	0.042	0.003	**0.954**	0.001	**0.798**	0.013	0.188	0.002
NEG Shrimp 17	SPW	JMA	0.000	0.042	**0.958**	0.000	0.001	**0.707**	0.290	0.002
NEG Shrimp 18	SPW	SPW	0.001	0.014	**0.985**	0.000	0.003	0.273	**0.716**	0.007
NEG Shrimp 19	SPW	SPW	0.002	0.001	**0.997**	0.000	0.042	0.018	**0.933**	0.007
NEG Shrimp 20	SPW	SPW	0.001	0.007	**0.992**	0.000	0.008	0.437	**0.555**	0.000
NEG Shrimp 21	SPW	SPW	0.008	0.004	**0.986**	0.001	0.208	0.171	**0.551**	0.070
NEG Shrimp 22	SPW	SPW	0.007	0.001	**0.992**	0.000	0.180	0.055	**0.751**	0.014
NEG Shrimp 23	SPW	SPW	0.001	0.001	**0.999**	0.000	0.007	0.033	**0.960**	0.001
NEG Shrimp 24	SPW	SPW	0.000	0.000	**1.000**	0.000	0.000	0.028	**0.971**	0.000
NEG Shrimp 25	SPW	SPW	0.004	0.002	**0.993**	0.001	0.052	0.095	**0.846**	0.007
NEG Shrimp 26	SPW	NOR	0.013	0.001	**0.824**	0.162	0.017	0.004	0.038	**0.942**
NEG Shrimp 27	SPW	SPW	0.004	0.008	**0.979**	0.009	0.041	0.126	**0.776**	0.057
NEG Shrimp 28	SPW	SPW	0.007	0.002	**0.990**	0.001	0.112	0.050	**0.828**	0.010
NEG Shrimp 29	SPW	NOR	0.004	0.000	**0.924**	0.072	0.022	0.001	0.212	**0.765**
NEG Shrimp 30	SPW	SPW	0.005	0.003	**0.976**	0.016	0.079	0.037	**0.751**	0.132
NEG Shrimp 31	SPW	SPW	0.003	0.005	**0.991**	0.001	0.021	0.201	**0.769**	0.009

NEG Shrimp 32	SPW	SPW	0.011	0.003	**0.985**	0.001	0.203	0.144	**0.645**	0.008
NEG Shrimp 33	SPW	JMA	0.005	0.026	**0.945**	0.024	0.029	**0.587**	0.273	0.111
NEG Shrimp 34	SPW	SPW	0.003	0.000	**0.996**	0.001	0.183	0.004	**0.798**	0.016
NEG Shrimp 35	SPW	SPW	0.004	0.000	**0.996**	0.000	0.128	0.002	**0.869**	0.002
NEG Shrimp 36	SPW	ICE	0.052	0.008	**0.837**	0.103	**0.504**	0.016	0.069	0.411
NEG Shrimp 37	SPW	SPW	0.000	0.000	**1.000**	0.000	0.000	0.001	**0.999**	0.000
NEG Shrimp 38	SPW	SPW	0.004	0.003	**0.993**	0.000	0.013	0.238	**0.749**	0.000
NEG Shrimp 39	SPW	SPW	0.002	0.002	**0.996**	0.000	0.030	0.109	**0.851**	0.010
NEG Shrimp 40	SPW	JMA	0.008	0.054	**0.937**	0.001	0.094	**0.680**	0.225	0.002

Supplementary Text S2: PCR Profiles & Microsatellite loci evaluation

The PCR profile for cod (from [48]) consisted of an initial denaturation step at 95 °C for 15 min, followed by 22 cycles of 95 °C for 30 s, 56 °C for 3 min and 72 °C for 1 min. The PCR reactions ended with a final elongation step of 60 °C for 30 min.

The PCR profile for redfish (from [15]) consisted of an initial denaturation step at 95 °C for 15 min, followed by 25 cycles of 95 °C for 30 s, 56 °C for 90 s and 72 °C for 1 min. The PCR reactions ended with a final elongation step of 60 °C for 45 min.

The PCR profile for shrimp (from [51]) consisted of an initial denaturation step at 95 °C for 15 min, followed by 35 cycles of 95 °C for 30 s, 56 °C for 3 min and 72 °C for 1 min. The PCR reactions ended with a final elongation step of 60 °C for 30 min.

MICRO-CHECKER (v. 2.2.3)[64] was used to identify large allele drop-out, null alleles, and stuttering scoring errors. Locus-wise deviation from HWE was analysed using GENEPOP (v. 4.2.1)[65] using exact tests[66], in addition to Linkage Disequilibrium (LD) identification between loci. In addition, BAYESCAN (v. 2.1)[67] and ARLEQUIN (v. 3.5.2.2)[68] were used to test loci neutrality with default settings. ARLEQUIN simulations examined the joint distribution of F_{ST} and heterozygosity under a hierarchical island model[69]. All results were judged for significance under the false discovery rate (FDR) approach[68] at the 5% level. Loci were not considered to be non-neutral unless both approaches reported them as such.

There was no evidence of large allele dropout in any loci. Several cases of stuttering and significant LD were reported, though these were deemed as non-genuine since genuine stuttering and LD are expected to affect all populations equally[71]. Loci were deemed to be subject to null alleles if null alleles were present in more than a single population. This applied to one locus of each species (cod: Gmo35, redfish: Spi6, shrimp: PbA104a). No loci deviated from HWE in more than a single population. No loci were consistently reported as non-neutral outliers under both approaches utilised.

Table S2. Microsatellite loci are shown in amplification multiplexes (MP's) and as utilised under both screening conditions and analyses (following: [15,48,51]).

Species	Condition 1	All loci
Cod	MP1: Gmo8, Gmo19, Gmo37, Tch11<br>MP2: Gmo2, Gmo3, Gmo34, Tch13, Gmo132	MP1: Gmo8, Gmo19, Gmo35, Gmo37, Tch11<br>MP2: Gmo2, Gmo3, Gmo34, Tch13, Gmo132
**Total**	**9**	**10**
Redfish	MP1: Sal1, Sal3, Sal4, Smen05<br>MP2: Spi4, Spi10, Smen10<br>MP3: Seb09, Seb25, Seb31, Seb33, Seb45	MP1: Sal1, Sal3, Sal4, Smen05<br>MP2: Spi4, Spi6, Spi10, Smen10<br>MP3: Seb09, Seb25, Seb31, Seb33, Seb45
**Total**	**12**	**13**
Shrimp	MP1: PbC8, PbC105, SD2-14<br>MP2: PbA110, PbC109, PbD9<br>MP3: PbA1, SD1-41, SD2-68, SD3-62	MP1: PbA104a, PbC8, PbC105, SD2-14<br>MP2: PbA110, PbC109, PbD9<br>MP3: PbA1, SD1-41, SD2-68, SD3-62
**Total**	**10**	**11**

Supplementary Text S3: Reference dataset evaluation

To test the power of assignment, the function `predict.dapc` in the R package *adegenet* was used to re-assign all reference samples back to their original *a priori* population clusters. The `x.val` function indicated the number of principle components to retain. ARLEQUIN was used to calculate pairwise F_{ST} values[72] between the reference populations. Only results for the all-loci condition of analysis are shown.

DAPC reassignment reported reference samples were successfully re-assigned to their original cluster (population) in 97% (cod) and 93% (redfish & shrimp) of cases. F_{ST} values ranged from 0.011 to 0.040 and were all highly significant (P <0.001) (Table S3).

Table S3. Pairwise F_{ST} values and P-values. The values in boldface are significant after false discovery rate control at P = 0.05. For abbreviations refer to Table S3.

Species					
		ICE	NCC	NEAC	
Cod	ICE	-	**0.000**	**0.000**	
	NCC	0.024	-	**0.000**	
	NEAC	0.014	0.023	-	
		NSH	EGS	IDP	
Redfish	NSH	-	**0.000**	**0.000**	
	EGS	0.031	-	**0.000**	
	IDP	0.037	0.040	-	
		NOR	SPW	ICE	JMA
Shrimp	NOR	-	**0.000**	**0.000**	**0.000**
	SPW	0.031	-	**0.000**	**0.000**
	ICE	0.011	0.019	-	**0.000**
	JMA	0.038	0.024	0.025	-

Supplementary Text S4: Reference population structure and details

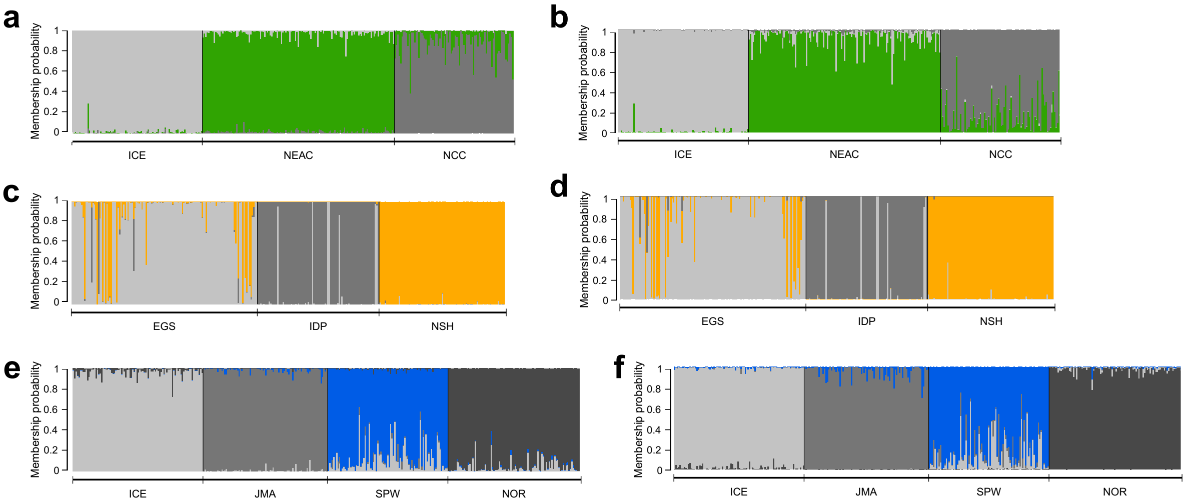

Fig. S4. STRUCTURE barplots showing Atlantic cod (*Gadus morhua*) (**a**, **b**), beaked redfish (*Sebastes mentella*) (**c**, **d**) and deep-sea shrimp (*Pandalus borealis*) (**e**, **f**) reference population membership probabilities (*q*) prior to the removal of individuals with a threshold of *q* lower/higher than 0.2/0.8. Barplots (**a**, **c** & **e**) utilised a reduced number of loci (Condition 1). Barplots (**b**, **d** & **f**) utilised all loci available regardless of potential scoring errors identified. For abbreviations refer to Table S4.

Table S4. Details of reference populations for each species, post removal of individuals with a threshold of *q* lower/higher than 0.2/0.8 and, under both analyses conditions. Abbr. = the abbreviated population name, *n* = sample size (number of genotyped individuals).

Species	Population	Abbr.	Condition 1 *n*	All loci *n*
Cod	Iceland	ICE	92	92
	Norwegian Coastal Cod	NCC	67	57
	North East Arctic Cod	NEAC	136	126
Redfish	Iceland Deep	IDP	80	80
	South East Greenland Slope	EGS	111	109
	Norway Shallow	NSH	91	90
Shrimp	Iceland	ICE	90	92
	Jan Mayen Island	JMA	87	87
	Norway	NOR	87	91
	Spitsbergen West	SPW	45	58

Supplementary Text S5: Evaluation of the impact of potential scoring errors on assignment

When using the datasets where loci subject to potential scoring errors (specifically, null alleles) were removed, individual assignment using STRUCTURE resulted in the same outcome as when analysing the data utilising all loci available, regardless of scoring errors. STRUCTURE barplots (Fig. S5) show that 100% of cod, 95% of redfish and 80% of shrimp tests resulted in the genetic assignment of individuals to a population within the Barents Sea, with a high membership probability (q >0.8) suggesting a high-likelihood that specimens of the three species found off Northeast Greenland originate from the Barents Sea.

DAPC clustering using datasets where loci with potential scoring errors were removed resembled the results when using all loci available (Fig. S5a,c,e). The NE Greenland group of all three species clustered closely with the same Barents Sea populations as indicated by the assignment testing (Fig. S5b,d,f). The 95% DAPC cluster ellipses between NEG and these population clusters overlapped considerably, though overlap was also evident between the reference population clusters, most significantly for the cod and shrimp clusters. The distance between the NE Greenland group clusters and clusters they were assigned to using STRUCTURE only differed for the shrimp data. Here, there is a greater distance between the NE Greenland shrimp group and Spitsbergen West shrimp group, than when analysed using all loci available.

Therefore, we suggest that the impact of potential null alleles on assignment was minimal and that utilising all loci available regardless of null alleles did not bias the assignment outcome.

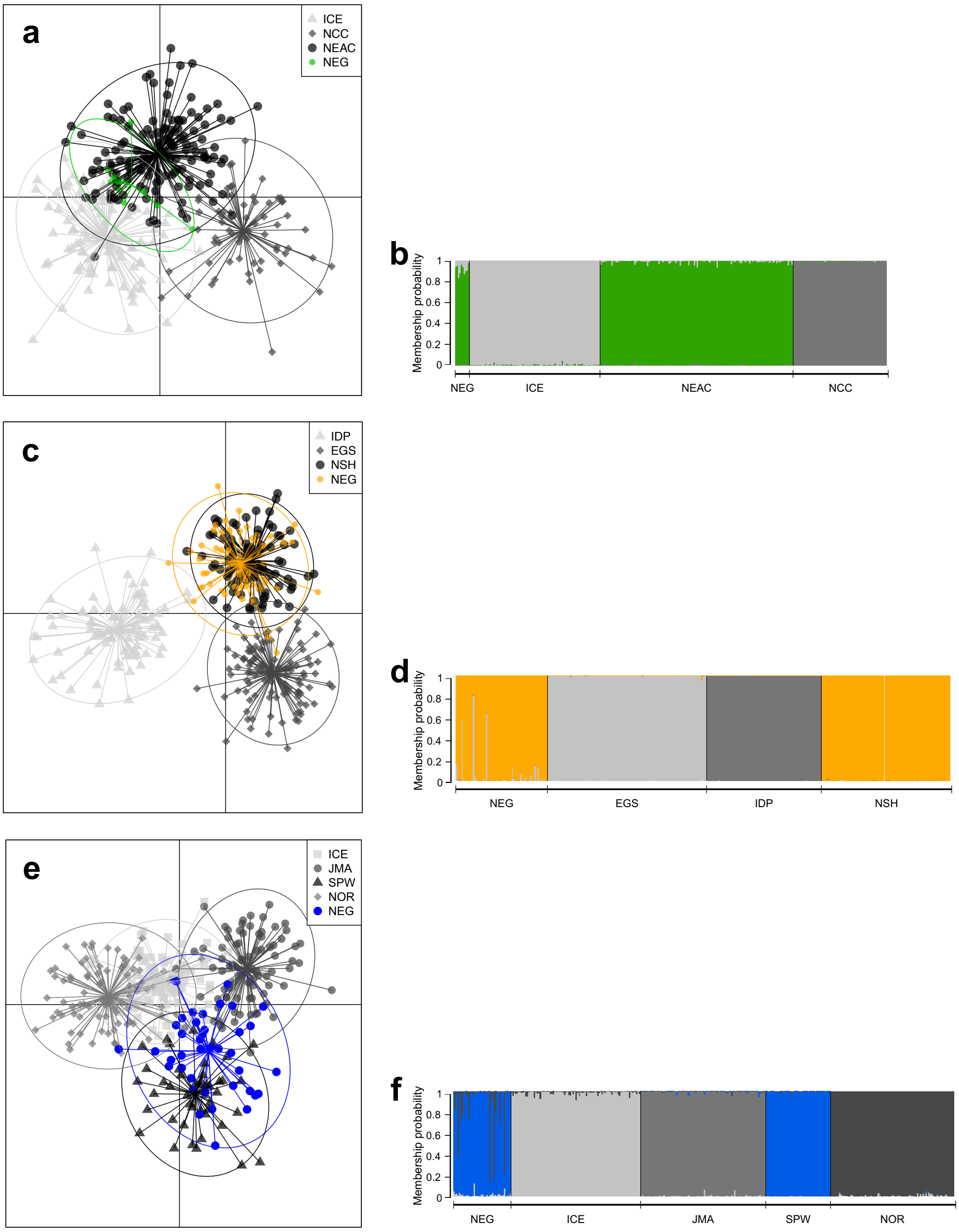

Fig. S5. Genetic analysis using a reduced set of loci that accounted for potential scoring errors, that we termed ‘Condition 1’, for Atlantic cod (*Gadus morhua*) (**a**, **b**), beaked redfish (*Sebastes mentella*) (**c**, **d**) and deep-sea shrimp (*Pandalus borealis*) (**e**, **f**) data. DAPC scatterplots (**a, c, e**) show how the NE Greenland groups relate to the reference populations of the Northeast Atlantic Ocean. DAPC cluster ellipses were set to contain 95% of genotypes. DAPC scatterplots explain 91% (**a**), 94% (**c**) and 99% (**e**) of the total variation observed. STRUCTURE barplots (**b**, **d**, **f**) show membership probabilities (*q*) for NE Greenland individuals based on the reference populations used. For abbreviations refer to Table S4.

Supplementary References

64. Van Oosterhout, C., Hutchinson, W.F., Wills, D.P. & Shipley, P. MICRO CHECKER: software for identifying and correcting genotyping errors in microsatellite data. *Molecular Ecology Resources* **4**(3), 535-538 (2004).
65. Rousset, F. Genepop'007: a complete re-implementation of the genepop software for Windows and Linux. *Molecular Ecology Resources* **8**, 103-106 (2008).
66. Guo, S.W. & Thompson, E.A. Performing the exact test of Hardy-Weinberg proportion for multiple alleles. *Biometrics*, 361-372 (1992).
67. Foll, M. & Gaggiotti, O. A genome-scan method to identify selected loci appropriate for both dominant and codominant markers: a Bayesian perspective. *Genetics* **180**(2), 977-993 (2008).
68. Excoffier, L. & Lischer, H.E. Arlequin suite ver 3.5: a new series of programs to perform population genetics analyses under Linux and Windows. *Molecular Ecology Resources* **10**(3), 564-567 (2010).
69. Beaumont, M.A. & Nichols, R.A. Evaluating loci for use in the genetic analysis of population structure. *Proceedings of the Royal Society B* **263**(1377), 1619-1626 (1996).
70. Benjamini, Y. & Yekutieli, D. The control of the false discovery rate in multiple testing under dependency. *Annals of statistics* **29**(4), 1165-1188 (2001).
71. Dormontt, E.E., Gardner, M.G., Breed, M.F., Rodger, J.G., Prentis, P.J. & Lowe, A.J. Genetic bottlenecks in time and space: reconstructing invasions from contemporary and historical collections. *PloS One* **9**(9), e106874 (2014).
72. Weir, B.S. & Cockerham, C.C. Estimating F-statistics for the analysis of population structure. *Evolution* **38**(6), 1358-1370 (1984).
